# The Hrs/Stam Complex Acts as a Positive and Negative Regulator of RTK Signaling during *Drosophila* Development

**DOI:** 10.1371/journal.pone.0010245

**Published:** 2010-04-21

**Authors:** Hélène Chanut-Delalande, Alain C. Jung, Magdalena M. Baer, Li Lin, François Payre, Markus Affolter

**Affiliations:** 1 Biozentrum der Universität Basel, Abteilung Zellbiologie, Basel, Switzerland; 2 Université de Toulouse, UPS, Centre de Biologie du Développement, Université Paul Sabatier, Toulouse, France; 3 CNRS, UMR5547, Centre de Biologie du Développement, Toulouse, France; Stockholm University, Sweden

## Abstract

**Background:**

Endocytosis is a key regulatory step of diverse signalling pathways, including receptor tyrosine kinase (RTK) signalling. Hrs and Stam constitute the ESCRT-0 complex that controls the initial selection of ubiquitinated proteins, which will subsequently be degraded in lysosomes. It has been well established *ex vivo* and during *Drosophila* embryogenesis that Hrs promotes EGFR down regulation. We have recently isolated the first mutations of *stam* in flies and shown that Stam is required for air sac morphogenesis, a larval respiratory structure whose formation critically depends on finely tuned levels of FGFR activity. This suggest that Stam, putatively within the ESCRT-0 complex, modulates FGF signalling, a possibility that has not been examined in Drosophila yet.

**Principal Findings:**

Here, we assessed the role of the Hrs/Stam complex in the regulation of signalling activity during *Drosophila* development. We show that *stam* and *hrs* are required for efficient FGFR signalling in the tracheal system, both during cell migration in the air sac primordium and during the formation of fine cytoplasmic extensions in terminal cells. We find that *stam* and *hrs* mutant cells display altered FGFR/Btl localisation, likely contributing to impaired signalling levels. Electron microscopy analyses indicate that endosome maturation is impaired at distinct steps by *hrs* and *stam* mutations. These somewhat unexpected results prompted us to further explore the function of *stam* and *hrs* in EGFR signalling. We show that while *stam* and *hrs* together downregulate EGFR signalling in the embryo, they are required for full activation of EGFR signalling during wing development.

**Conclusions/Significance:**

Our study shows that the ESCRT-0 complex differentially regulates RTK signalling, either positively or negatively depending on tissues and developmental stages, further highlighting the importance of endocytosis in modulating signalling pathways during development.

## Introduction

Receptor Tyrosine Kinase (RTK) signalling pathways regulate a wide range of biological processes and functions, including cell survival, growth, differentiation and migration [1]. Regulating levels of RTK signalling activity in time and space is an important issue since its deregulation leads to developmental defects and pathologies, including human cancers [2]. Besides ligand availability, the levels of RTK signalling are strongly influenced by endocytic processes [3], [4], [5]. The Endosomal Sorting Complex Required for Transport (ESCRT) machinery comprises four protein complexes (ESCRT-0 to III) that sequentially act to target activated RTK to multivesicular bodies (MVB), en route to the lysosome for degradation, thus promoting signalling downregulation. After endocytosis from the plasma membrane into early endosomes, ubiquitinated RTKs are bound by the ESCRT-0 complex composed of Hrs (Hepatocyte growth factor-regulated tyrosine kinase substrate) and Stam (Signal Transducing Adaptor Molecule) proteins [6], [7]. The Hrs/Stam complex thus plays a key role in sorting cargo proteins either for degradation or recycling to the plasma membrane [8], [9]. Indeed, Hrs selects ubiquitinated cargos for lysosomal degradation [7], [10], [11], recruiting components of other ESCRT complexes (I, II & III), which in turn send cargos to MVB, late endosomes and finally to lysosomes [12], [13], [14]. Results from numerous studies in cultured cells have shown that the Hrs/Stam complex is required for attenuation of Epidermal Growth Factor Receptor (EFGR) signaling and suggested a general function in RTK down regulation. However, the Stam protein was reported to interact with deubiquitination enzymes [15], [16], raising the possibility that it can also contribute, in interaction with or independently of Hrs, to disengagement of the cargo from the degradation pathway. Therefore, the full deciphering of the role of Hrs and Stam requires *in vivo* analysis of their respective function during the different processes that are regulated by signalling pathways during development.

Previous works in *Drosophila* have clearly show that Hrs indeed act to attenuate EGFR signalling during embryogenesis [17]. Embryonic *hrs* mutant cells display more EGFR signalling activity and accumulate activated receptors and ubiquitinated proteins in enlarged endosomes [17], [18], [19], [20], [21]. In addition, *hrs* mutant cells show a marked accumulation of other activated RTK, including PDGF/VEGF receptors, as well as other types of signalling receptors such as Patched and Smoothend (two members of the Hedgehog pathway), Notch, and Thickveins (a type-I serine-threonine kinase receptor for the TGF-β ligand Dpp) [19]. Strikingly, it has been shown that loss of *hrs* actually increases Dpp signalling in ovarian follicle cells and wing disc cells [19]. Furthermore, Hrs appears necessary for efficient JAK/STAT signalling during drosophila oogenesis [18]. Nevertheless, the consequences of enhanced signalling activity on developmental process due to the lack of *hrs* could be weak on development as shown during oogenesis where two RTK signalling pathways, EGFR and PDGF/VEGF are essential for border cell migration [19]. Despite an enhancement of these two pathways in *hrs* mutant border cells, the cell migration process is not affected. Jekely et al have shown that the total level of RTK activity is not critical for border cell migration but correct migration requires a restricted subcellular localisation of activity in the cell[22]. Those analyses brought new insights on mechanisms controlling signalling activity required for efficient cell migration. Altogether, these data reveal the complexity of the mechanisms relying on Hrs activity to regulate various signalling activities *in vivo*, suggesting that whereas required for down regulation of RTK, Hrs is also necessary for efficient signalling of other receptors. Whether these differential activities involve the full ESCRT-0 complex, or correspond to Stam-independent function of Hrs, remains yet to be tested.

**Figure 1:**
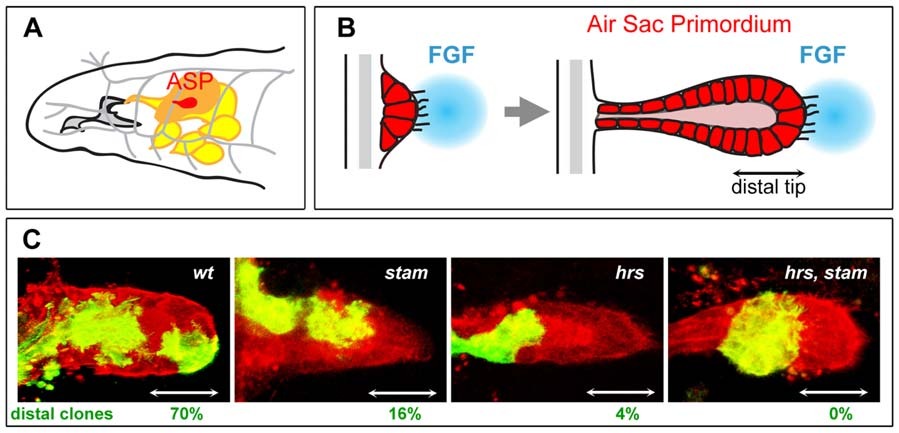
*stam* and *hrs* are required for tracheal cell migration in the air sac primordium. A. Schematic representation of the anterior part of a *Drosophila* third instar larva. The air sac primordium (ASP) (red) buds from the transverse connective branch (in grey) and is attached to the wing imaginal disc (orange). The tracheal system is drawn in grey and imaginal discs other than the wing disc are colored in yellow. B. Model for the formation of the air sac primordium during larval development. Tracheal cells divide and migrate during ASP formation. Migration occurs under the control of the FGFR signalling pathway. Tracheal cells at the distal tip of the primordium are extending filapodia in the direction of the FGF ligand source (blue). Double arrow indicates the position of ASP distal tip. C. Migration behaviour of *wild type*, *stam*, *hrs* and *stam hrs* mutant cells. Confocal micrographs of the ASP of a *Drosophila* third instar larva are shown. All tracheal cells are labelled in red (RFP-moesin) and MARCM clones are labelled in green (mCD8-GFP). The *FRT40A* chromosome was used as a wild-type control. MARCM clones were induced for *stam*, *hrs* and *stam*, *hrs*. Scale bar: 15 µm. White double arrows indicate the position of ASP distal tip. Percentages of distal clones are indicated for each genotype tested. Note the strong effect of mutations in *hrs* and *hrs, stam* on cell migration. For each genotype, more than 20 clones were scored.

We have recently isolated the first mutations inactivating the *Stam* gene in *Drosophila* in a genetic screen aiming at identifying novel molecules involved in the formation of the larval tracheal system [23]. The *Drosophila* tracheal system develops during embryogenesis through the formation of branches arising from epithelial sacs containing approximately 80 cells in ten adjacent segments of the embryo. Through cell migration, rearrangement and intercalation, a complex network of interconnected epithelial tubes is formed to permit gas exchanges throughout the embryo. The development of the tracheal system has proven to be a model of choice to study branching morphogenesis and has been useful to genetically dissect the FGF pathway [24], [25], [26]. The FGF ligand, Branchless (Bnl), binds and activates the FGF receptor tyrosine kinase Breathless (Btl), which is expressed in all tracheal cells and triggers cell migration upon activation [27], [28]. However, despite extensive genetic analyses, relatively little is known about how signalling is finely tuned during embryonic tracheal development. Indeed, genes linked to RTK signalling display pleiotropic functions during embryogenesis; the observed phenotypes, which affect several tissues as well as developmental processes, often hinder interpretations with regards to their role in trachea. Moreover, due to a strong maternal contribution to the egg during oogenesis, the function of many genes cannot be assessed during embryogenesis using zygotic loss-of-function analysis. Some of these problems can be circumvented by studying the development of tracheal structures formed at later stages, in larvae. During the third larval instar, tracheal cells of the transverse connective in the second thoracic segment bud out and form the primordium of a so-called dorsal air sac, or Air Sac Primordium (ASP) (Fig. 1A). The development of ASP relies on both cell proliferation and cell migration [29], [30], [31]. Genetic analyses of mosaic animals, using the MARCM (Mosaic Analysis with a Repressible Cell Marker) technique [32], have shown that during air sac morphogenesis, the FGFR pathway controls tracheal cell migration at the tip of the outgrowths (Fig. 1B), while EGFR signalling triggers cell survival and proliferation in the stalk [32], [33]. Moreover, the Ras/MAPK cascade, commonly used to transduce RTK signals, is required for both tracheal cell migration and survival. However, the Pointed ETS transcription factor was found to be specifically required downstream of the Ras/MAPK cassette for cell migration [33].

To better understand the molecular mechanisms controlling air sac morphogenesis, we previously carried out a genetic mosaic screen and identified the *stam* gene as being essential for efficient tracheal cell migration in the ASP [23]. In the absence of Stam, mutant cells are unable to reach the distal tip of the ASP, showing that, like FGF signalling, Stam is required for efficient cell migration. These data therefore open the intriguing hypothesis that, during ASP formation, the activity of Stam, and putatively that of the ESCRT-0 complex, may be required for efficient FGF signalling. Here we explore this possibility and show that both Stam and Hrs act as positive regulators of FGF-mediated RTK activity to control cell migration in the ASP. Mutant cells lacking Stam, Hrs, or both, display abnormal accumulation of the FGF receptor Breathless (Btl) in enlarged vesicles, likely representing early endosomes. This is accompanied by a reduced expression of *pointed*, showing that the lack of ESCRT-0 activity specifically impairs efficient FGF signalling in ASP cells. In addition, we show that Stam and Hrs are required for the FGF-mediated formation of fine cytoplasmic extensions in terminal tracheal cells, further illustrating the importance of the ESCRT-0 complex in the positive regulation of FGF signalling. In contrast, we find that, like Hrs, Stam is required for down-regulation of EGFR signalling in the embryo. However, our data provide evidence to show that the ESCRT-0 complex is required for full activation of EGFR signalling during wing development. Taken together, our results indicate that, depending upon signalling pathways and the cellular context, the ESCRT-0 complex promotes either efficient activation or down-regulation of RTK pathways, shedding novel light on the importance of the endocytic control of signalling activities *in vivo*.

## Materials and Methods

### *Drosophila* stocks and genetics

The following fly strains were used: *stam^2L2896^*, *stam^2L3297^* [23] and *hrs^D28^* (gift from H. Bellen). The *stam^2L2896^* line harbours a non sense mutation in codon 6 suggesting that this *stam* allele represents a null allele. In addition, an Ala^505^ to a Thr substitution was found in the *stam^2L2896^* line. The *stam^3297^* line also carried two mutations in the *stam* locus: a Phe^428^ to Ser mutation and a Tyr^528^-to-His substitution. This latter change may be the one affecting Stam protein function since Tyr^528^ is a favourable environment for phosphorylation. The *hrs, stam* recombinants were generated *via* standard genetic procedures. As a wild type control, we used a FRT40A line. The following strains were used: *UAS-stam* to rescue the migration phenotype [23], *UAS-Rab5-GFP* (gift from M. Gonzalez Gaitan) to detect early endosomes and *UAS-Btl-GFP* to follow the localisation of the FGFR/Btl protein [29]. We also used the *bnl^P1^* mutant [28] and the enhancer trap lines *argos^W11^* and *pointed (l(3)7825)* [34].

To obtain embryos lacking the maternal contribution of *stam*, progeny of adult females of the following genotype *hsFLP/+; stam FRT40A/P[ovoD1], P[ovoD1]FRT40A* received one heat pulse at 38°C, for 1 hr during first-instar larval development. For *hrs* and *stam, hrs* mutations, similar procedures were followed using females from genotypes *hsFLP/+; hrs FRT40A/P[ovoD1],P[ovoD1]FRT40A and hsFLP/+; stam, hrs FRT40A/P[ovoD1], P[ovoD1]FRT40A*, respectively.

### Clonal analysis using the MARCM technique

#### Induction of MARCM clones in the tracheal system and in the air sac primordium

Clones in the air sacs were obtained according to procedures previously described in Cabernard and Affolter, (2005) and using the following MARCM lines: y,w,*hsFlp; tubGal80, FRT40A/CyO; btlenhancer-mRFP1moe, btl-Gal4, UAS-mCD8-GFP/TM6C* to visualize the clones in the ASP and *y,w,hsFlp; tubGal80, FRT40A/CyO; btlenhancer-mRFP1moe, btl-Gal4/TM6C* to detect the clones in the ASP with either *Rab5-GFP* or *Btl-GFP*.

MARCM females were crossed to males carrying the mutation of interest. The progeny of these crosses was staged, and embryos that had reached 4–6 hr of development were heat-shocked for 1 h at 38°C. They were further grown at 25°C until the third instar larval stage. GFP-positive larvae were sorted using a GFP stereomicroscope (Leica MZFLIII) and wing imaginal discs were dissected [33]. For terminal cell analysis, GFP positive larvae were heat-shocked in a heat-block for 30 seconds at 70°C and mounted in glycerol.

#### Induction of clones in the wing imaginal discs and pupal wings

The following lines (gift from B. Bello) were used to induce MARCM clones:

- *y,w,hsFlp;FRT40A,tub-Gal80/CyoActinGFP; Tub-Gal4, UAS-mCD8-GFP/TM6,Tb,Hu* to detect clones in the wing imaginal discs and pupal wing
- *y,w,hsFlp;FRT40A,tub-Gal80/CyoActinGFP; Tub-Gal4/TM6,Tb,Hu* to visualise the clones in the wing imaginal discs with *UAS-Rab5-GFP*.

MARCM females were crossed to mutant males and first instar larvae of the progeny received one pulse of heat shock at 38°C for 1 h.

### Staining, electronic microscopy experiments and imaging

Immunostaining of dpERK in embryos lacking the maternal contribution of *hrs* and/or *stam* genes was performed using standard procedures (anti dpMAPK, Sigma, 1/500).

To visualise *pointed* and *argos* expression, lacZ staining was performed on wing imaginal discs or dissected pupal wings: Anti mouse Alexa 568 and 633 (1∶500 Molecular Probes, Invitrogen) were used as secondary antibodies to detect the staining.

Confocal pictures were made using a Leica TCS SP2 microscope. Pictures from the entire ASP were made to detect the position of the clones. To visualise the localisation of Btl-GFP and Rab5-GFP, the pictures correspond to single section of the ASP. To count the branching points of terminal cells, pictures of all branches were done.

Garland cell preparation was performed according to procedures described previously [17], [35]. Sections were photographed with a Leo 510 (Zeiss) microscope (Zentrum Mikroskopie der Universität Basel). Endosome areas were measured using ImageJ software for each genotype.

## Results

### The Hrs/Stam complex is essential for FGF-mediated tracheal cell migration in the air sac primordium (ASP)

Using the MARCM technique, we have recently shown [23] that the loss of Stam impairs the migration of air sac primordium (ASP) cells; with only 16% of mutant clones detected at the ASP tip (Fig. 1C). Interestingly, migration of cells at the tip of the air sac primordium requires FGFR signalling activity, since mutant cells lacking components of the pathway (like *btl* mutant clones, for instance) are unable to reach the distal tip of the ASP.

Since Stam is known to be a direct partner of Hrs within the ESCRT-0 complex [6], we tested a putative role of *hrs* during tracheal cell migration in the ASP using the *hrs^D28^* null allele. Cell migration is strongly affected in *hrs* mutants, as only 4% of *hrs* clones are able to colonize the distal tip of the ASP (Fig. 1C). Cells lacking both *hrs* and *stam* exhibited a severe migration phenotype; double mutant clones were never detected at the distal tip of the ASP (Fig. 1C). Such a strong migration phenotype was previously only observed following the inactivation of genes encoding essential components of the FGFR signalling pathway [33]. In addition to the failure to locate at the tip of outgrowing ASP, *hrs, stam* double mutant clones often exhibited a reduced number of cells compared to *stam* or *hrs* single mutant clones suggesting that the lack of ESCRT-0 function also affects cell proliferation to a certain extent. Taken together, these results show that *hrs* and *stam* are required for the FGF-dependent migration of tracheal cells during larval development.

### Btl/FGFR accumulates in enlarged vesicles in *stam* and *hrs* mutant air sac cells

**Figure 2:**
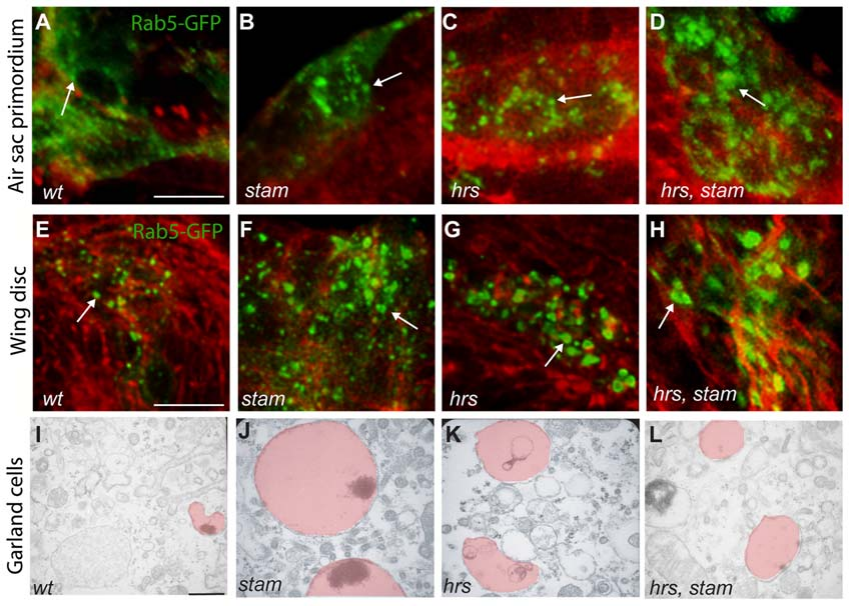
*hrs* and *stam* mutants display enlarged early endosomes in *Drosophila*. A–D. Close-up pictures of *wild type*, *stam*, *hrs* and *hrs, stam* mutant cells induced with the MARCM system in the dorsal ASP. Scale bar: 15 µm. Mutant clones are visualised by the expression of Rab5-GFP to detect early endosomes. Note the large size of early endosomes in *stam* (B), *hrs* (C) and *hrs, stam* (D) mutant cells compared to *FRT40A* cells used as a wild type control (A). The tracheal cells were labeled with RFP-moesin (red). Arrows indicate early endosomes stained with GFP. Pictures shown are single scan sections. E–H. MARCM clones mutant for *hr*s, *stam* and *hrs, stam* in the wing imaginal disc of third instar larvae. Scale bar: 15 µm. Mutant cells express the Rab5-GFP fusion protein. As in the ASP, early endosomes are enlarged in *stam* (F), *hrs* (G) and *hrs, stam* (H) mutant cells compared to *FRT40A* cells used as a control (E). The cells were detected with phalloidin to follow F-actin and the shape of the cells. Arrows indicate early endosomes stained with GFP. Pictures shown are single scan sections. I–L. HRP uptake experiment in Garland cells of *wild-type*, *stam, hrs* and *hrs, stam* larvae. I. In wild-type Garland cells, many small endosomes are observed (highlighted in light red). Scale bar equals 460 nm. Endosomes from *stam* and *hr*s mutant larvae are dramatically enlarged (J–L), especially in *stam* mutants (J), compared to *FRT40A* larvae (I).

Since the ESCRT-0 complex is known to promote the endocytic degradation of many signalling receptors [17], [19], we analyzed the consequences of the loss of Stam and Hrs on endosome formation in ASP cells. We induced MARCM clones carrying either *stam* single mutations or *hrs, stam* double mutations. Clones were visualised *via* the expression of the fusion protein Rab5-GFP, Rab5 being a marker for early endosomes [36]. In wild type cells, Rab5-GFP-positive early endosomes were detected as small dot-like structures in the cytoplasm (Fig. 2A). In *stam* and *hrs* single mutant, as well as in *stam, hrs* double mutant cells, Rab5-GFP accumulates in larger dots (Fig. 2B–D), suggesting that early endosomes were enlarged in the absence of ESCRT-0 activity. Interestingly, this early endosome size defect was not restricted to tracheal cells, since we observed a similar phenotype in wing imaginal cells lacking *stam*, *hrs* or both (Fig. 2E–H). To better characterise the role of ESCRT 0 complex, we performed transmission electron microscopy (TEM) analyses of Garland cells, which are known to have a rapid rate of fluid phase endocytosis [35]. HRP (Horse Radish Peroxidase) uptake experiments allow the detection of the lumen and endosome membranes. Ultrastructural analysis of wild-type Garland cells has demonstrated that endosomes undergo maturation *via* a step of membrane invagination [17] (Fig. 2I). We measure the endosome area and we showed that in wild type Garland cells, the average area is 0,071+/−0,048 mm^2^ (Table 1). Consistently with a previous report [17], we observed that the endosomes of Garland cells lacking *hrs* are 2,7 fold larger than wild type ones (average area 0,199+/−0,110 mm^2^) (p<0,005) and do not exhibit HRP staining in the lumen, a phenotype likely due to defective membrane invagination (Fig. 2K and Table 1). Our analyses further revealed that *stam* mutant cells displayed even larger endosomes (1,192+/−0,557 mm^2^, 16,6 fold compared to wild type) (p<0,005) than those observed in the absence of *hrs* (Fig. 2J and Table 1). Garland cells lacking simultaneously *hrs* and *stam* mutations exhibited endosomes of the size of those observed in *hrs* mutant cells (0,216+/−0,108 mm^2^) (3 fold larger than wild type (p<0,005) (Fig. 2L and Table 1), suggesting that while *hrs* and *stam* are both involved in endosome maturation, they may have partly distinct roles during this process [7].

**Table 1:** Average area of endosomes analyzed in Garland cells from *wt, stam, hrs* and *hrs, stam* larvae.

genotype	Average area (mm^2^)	N
*wt*	0,071+/−0,048	47
*stam*	1,192+/−0,557	16
*hrs*	0,199+/−0,110	38
*hrs, stam*	0,216+/−0,108	19

**Figure 3:**
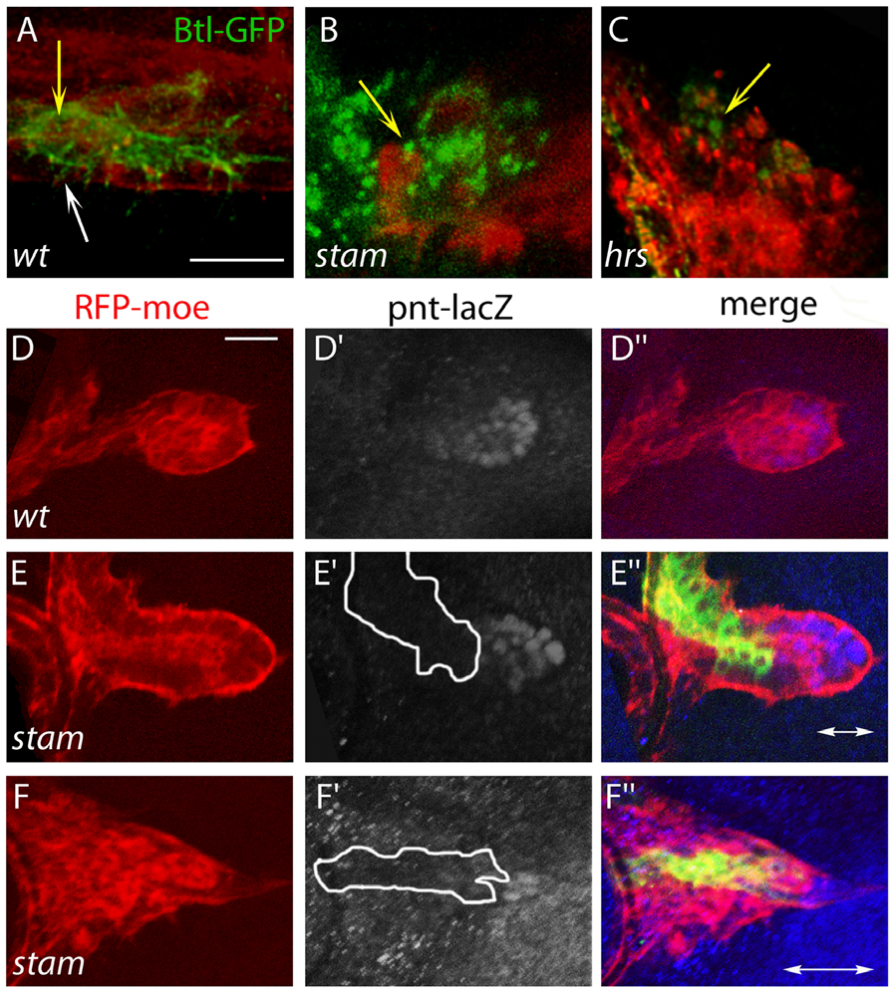
*stam* is required to properly localise FGFR/Btl and fully activate FGFR signalling. A–C. Localisation of Btl in wild type and mutant tracheal cells. High magnification pictures of wild type (A), *stam* (B) and *hrs* (C) MARCM mutant cells in the ASP. Scale bar equals 15 µm. Mutant cells were visualised *via* the expression of *UAS-btl-GFP* (green). The tracheal cells were visualised with RFP-moesin (red). White arrow indicates the presence of Btl at the cell membrane in wild type cells while yellow arrows indicate Btl as dotted structures. The dotted structures are dramatically enlarged in *stam* and *hrs* mutant cells as compared to *wild type* cells. D–F. *pointed* expression in wild type and *stam* mutant tracheal cells. Scale bars: 15 µm. *pointed* expression is restricted to the distal part, the tip, of a wild type ASP (D–D″). In a *stam* mutant clone located at the proximal part of the ASP, *pointed* expression is unchanged (E–E″). When the *stam* clone is positioned close to or at the distal tip of the ASP: *pointed* expression is lost (F–F″). Dotted lines showed the position of the *stam* mutant cells in the ASP (E′, F′). Arrows indicate the distal tip of the ASP (E″, F″).

Having shown that the absence of the ESCRT-0 complex leads to endosomal defects in different tissues, we next examined whether it affected the distribution of the FGF-R Btl in ASP cells. We performed mosaic analyses using a Btl-GFP fusion protein, specifically expressed in clones of control, *stam* or *hrs* mutant cells. In wild type cells, Btl-GFP mostly accumulated at the cell membrane (Fig. 3A, white arrow) and also as fine dotted structures in the cytoplasm, often lined up along the cell membrane (Fig. 3A, yellow arrow). In *hrs* and *stam* mutant clones, Btl-GFP was no longer detected at the membrane but was found in severely enlarged cytoplasmic vesicles (Fig. 3B–C). These data show that the lack of ESCRT-0 activity in tracheal cells of the ASP leads to the accumulation of the Btl receptor in enlarged vesicles, likely corresponding to the abnormal early endosomes revealed by Rab-5 staining.

### Stam and Hrs positively regulate FGFR activity in tracheal cells

Together with a reduced migration at the distal tip of the ASP, the abnormal accumulation of Btl in cells lacking Stam or Hrs suggests that ESCRT-0 regulate the FGF pathway. However, both the lack and the excess of FGF activity blocks tracheal cell migration. To determine a possible influence of the ESCRT-0 on FGF activity in ASP cells, we analysed the expression of the *pointed* (*pnt*) gene, an early target of the pathway that is specifically required for tracheal cell migration in the ASP [33]. Using a *pnt-lacZ* reporter line, we detected accordingly a staining in the distal part of the ASP in wild-type cells (Fig. 3D–D″). This is in agreement with the view that tracheal cells of the ASP distal tip actively migrate under the influence of the FGFR signalling. In *stam* mutant clones located in the proximal part of the ASP, we did not detected modification of the *pointed* staining, not even in cells at the most distal part of the clones, indicating that the expression of *pnt* is not upregulated under conditions in which activated Btl accumulates in enlarged endosomes (Fig. 3E–E″). Furthermore, when *stam* mutant cells were located close to the distal tip of the ASP, they did not express *pnt* despite the fact that they are in the vicinity to the source of the FGF ligand (Fig. 3F–F″). Hence these results are in line with *stam* being required for *pnt* activation and thus efficient FGFR signalling in the migrating cells of the ASP. Due to their absence at the tip of the ASP, we could not use however *pnt* expression as readout for FGFR activity in *hrs* single mutant and *hrs, stam* double mutant backgrounds.

**Figure 4:**
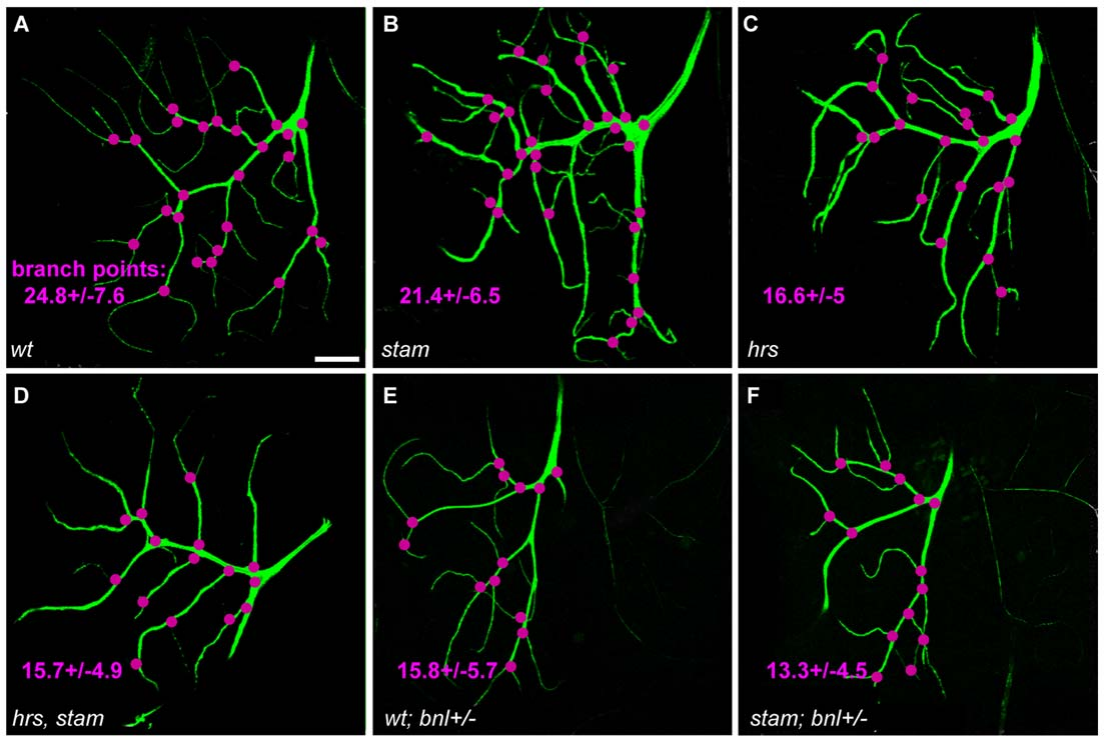
*stam* and *hrs* are required for the efficient formation of fine cytoplasmic extensions in tracheal terminal cells and interact with FGFR signalling. Confocal micrographs of MARCM wild type and mutant dorsal terminal cells. Scale bar: 50 µm. The clones are visualised using UAS-mCD8-GFP. FRT40A line was used as a control (A). MARCM clones were induced for *stam* (B), *hrs* (C), *hrs, stam* (D). (E–F). The FRT40A and *stam* MARCM terminal cells with altered FGFR signalling. *bnl+/−* corresponds to a larvae heterozygous for *bnl*. Branch points (visualised in pink) were counted for mutant clonal cells for each genotype. The average number of branch points is given for each genotype.

To further characterize the function of the Stam/Hrs complex in the regulation of FGFR signalling, we therefore analysed the formation of fine cytoplasmic extensions by terminal tracheal cells (TCs), a process which is also known to rely on Btl function. TC extensions are the finest branches of the tracheal system, which directly provide oxygen to individual cells. During larval development, the number of TC branches changes according to the needs of tissues for oxygen and this process is controlled, in a dose-dependent manner, by levels of FGFR signalling [37], [38], [39]. We thus analysed whether *stam* and *hrs* influenced the formation of TC extensions. We generated MARCM TC and counted the number of branch points formed by these mutant cells, taking into account both thick (primary) and thin (secondary and tertiary) projections [37]. To reliably compare the different genetic backgrounds, we focused on dorsal TCs. In wild type TCs, the average number of branching points was 24.8±7.6 (Fig. 4A and table 2). In *stam* mutant cells, this number decreased to 21.4±6.5 (p<0.001) (Fig. 4B). *hrs* mutant and *hrs, stam* double mutant cells displayed a much reduced capacity to sprout since we observed 16.6±5.0 (p<0.001) and 15.7±4.9 (p<0.001) branching points, respectively (Fig. 4C–D and table 2). These results thus indicate that *stam* and *hrs* are necessary for efficient TC sprouting.

**Table 2:** Number of branching points of *hrs* and *stam* MARCM terminal cell clones in different FGFR signalling activity backgrounds.

Genotype	Branching points	N
*wt*	24.8±7.6	209
*stam*	21.4±6.5	207
*hrs*	16.6±5.0	200
*hrs, stam*	15.7±4.9	99
*wt; bnl^−/+^*	15.8±5.7	86
*stam; bnl^−/+^*	13.3±4.5	103
*hrs; bnl^−/+^*	14.6±4.2	66
*hrs, stam; bnl^−/+^*	11.2±4.7	23

As previously described [28], lowering levels of FGF activity leads to a similar reduction in the number of TC extensions. For example, the strong *bnl^P1^* allele of *branchless* in heterozygous condition leads a clear decrease in the number of branching points (15.8±5.7), when compared to wild-type controls (p<0.001) (Fig. 4E–F and table 2). In terminal cells heterozygous for *bnl*, the absence of *stam* further increased the observed defects, with 13.3±4.5 branching points per cell. Although we recovered very few larvae carrying MARCM clones in the tracheal system for both *hrs* single and *hrs, stam* double mutants, these mutations also enhanced the phenotype resulting from lowering levels of FGF signalling.

Taken together, our results show that the function of the ESCRT-0 complex is required for efficient FGFR signalling in different aspects of tracheal cell development, including migration at the distal tip of the ASP and the formation of cytoplasmic projections in terminal cells.

### The ESCRT-0 complex displays opposite roles in the regulation of EGFR signalling throughout development

While our results show that the ESCRT-0 complex can positively regulate RTK activity during development, Hrs is required for down-regulating the EGFR activity in embryos [17]. We therefore tested whether *stam* acts similarly to *hrs* regarding EGFR signalling during embryogenesis. We used an antibody specific of the double phosphorylated form of ERK (dp-ERK) since EGFR signalling triggers ERK/MAPK phosphorylation in the early embryo [40]. In order to circumvent the maternal contribution of *stam* and *hrs*, we obtained embryos from germ-line clones lacking maternal and zygotic expression of *stam*, *hrs*, or both, and compared the results to wild type embryos.

**Figure 5:**
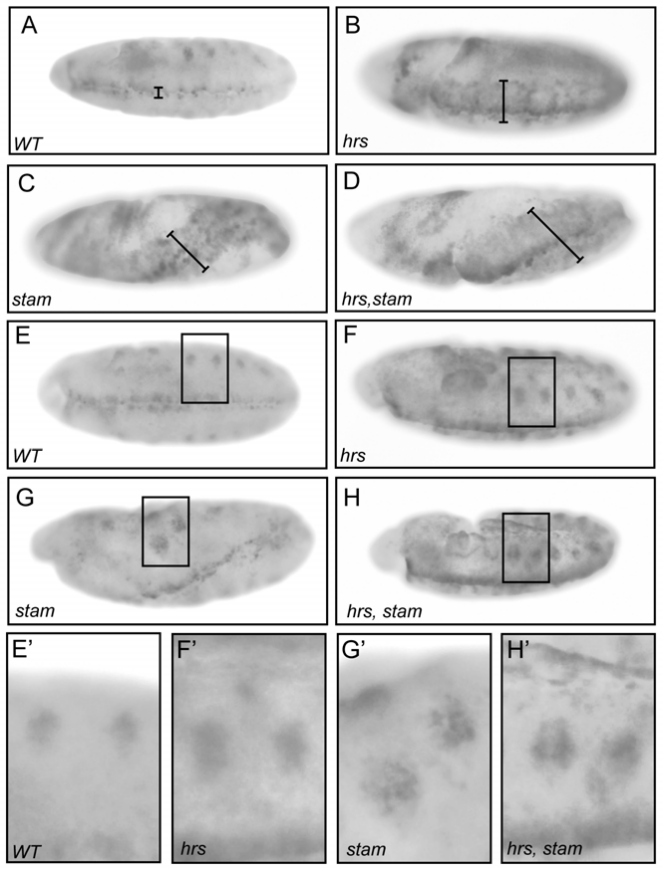
*stam* and *hrs* downregulate EGFR signalling activity during embryogenesis. Diphospho-ERK (dpMAPK) antibody staining in *wild type*, *hrs*, *stam* and *hrs, stam* maternal-zygotic loss-of-function embryos (A–H′). At stage 10, *hrs*, *stam* and *hrs, stam* mutants display enhancement of the dp-ERK staining (B–D), due to an expansion of their ventral fate (see black bar length) compared to a wild type embryo (A). Mutant embryos also display an expansion of the staining in tracheal placodes (F–H) compared to wild type (E). Close up views of the dp-ERK staining in the tracheal placodes (E′–H′).

During embryogenesis, the EGFR pathway is essential to pattern the neuroectoderm. dpERK is observed in three to four rows of cells, on each side along the ventral midline in wild-type embryos, when the Spitz ligand activates the EGFR pathway (Fig. 5A). Compared to wild-type embryos, we detected an expansion of dpERK staining in the ventral ectoderm in embryos lacking the maternal contribution for *hrs* (Fig. 5B) in agreement with the study of Lloyd et al (2002). In embryos lacking *stam* and those lacking simultaneously *stam* and *hrs* (Fig. 5C–D), we observed a similar enhancement of dpERK staining, attesting for elevated levels of EGFR signalling. Moreover, mutant embryos showed morphological defects due to aberrant folding of the embryo during germ band elongation, defects that are likely due to the expanded EGFR activation. We also monitored dpERK staining in stage 10 embryos, when the EGFR signalling pathway becomes required for the invagination of tracheal cells and the formation of tracheal placodes. At this stage of embryonic development, dpERK staining is detected in tracheal pits [40]. When compared to wild type, embryos lacking *hrs* or *stam*, or both *stam* and *hrs* display a clear expansion of the dpERK staining around the tracheal pits for all mutant conditions (Fig. 5E–H′). Therefore, the data indicate that Stam and Hrs play similar role in the attenuation of EGFR signalling throughout embryonic development.

**Figure 6:**
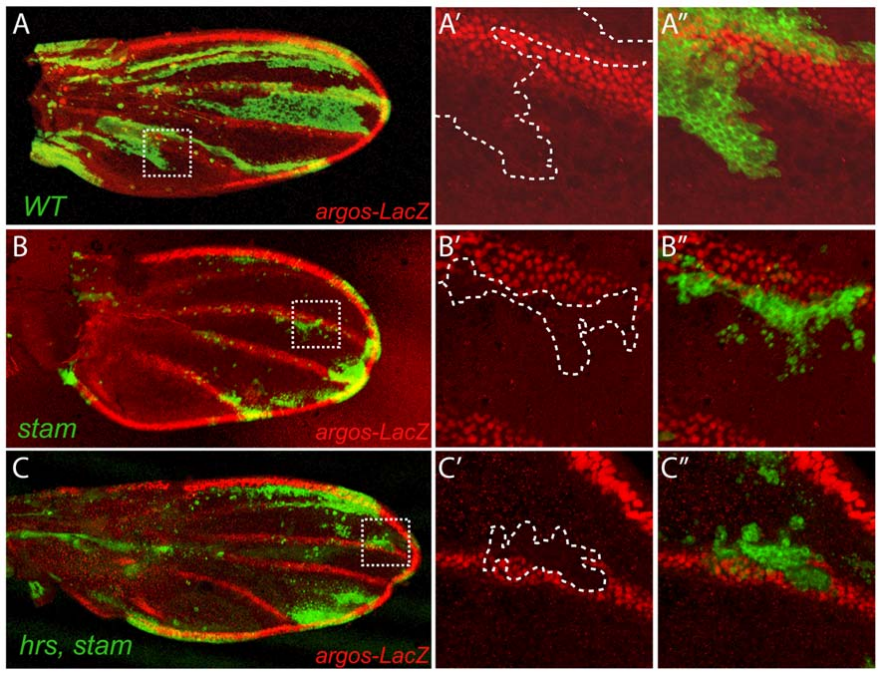
*argos* expression is strongly reduced in *stam*, *hrs* and *hrs, stam* mutant cells in pupal wings. *argos* expression was analysed in *wild type* (A), *stam^2L2896^* (B) and *hrs, stam* (C) MARCM clones visualized with mCD8-GFP (green) in pupal wings (24–30 hrs APF) using a *argos*-*LacZ* enhancer trap line. White dotted squares (in A–C) indicate the position of close-up pictures. White doted lines (in A′–C′) indicated the position of the clones in the close-up pictures. The *argos* staining (red) is absent in stam and *hrs, stam* mutant cells.

These results also suggested that the ESCRT-0 complex acts to down-regulate EGFR signalling throughout development. To further test this conclusion, we then extended our analysis to adult wing development, for which the role of EGFR regulatory cascade is well established [41]. To analyse a putative influence of *hrs* and *stam* on the regulation of EGFR signalling during wing patterning, we followed the expression of *argos*, a target gene of the EGFR pathway [42]. The vein differentiation process that initiates during the 3^rd^ larval instar proceeds into the pupal stage and, during the entire process, *argos* is activated in vein tissues (Fig. 6A). Cells that lack *stam* displayed a strong reduction of the *argos* expression, when clones of mutant cells were located in the vein tissue (Fig. 6B) Although *stam^2L2896^, hrs* mutant clones are tiny and do not permit to analyse *argos* expression, we also found a strong reduction of *argos* in vein cells carrying simultaneously the *stam^2L3297^* and *hrs^D28^* mutations (Fig. 6C). In contrast to its role in EFGR attenuation in embryonic tissue, these results thus support that the Hrs/Stam complex is required to efficiently activate EGFR signalling in the developing wing veins.

## Discussion

In this study, we have analyzed roles of the endocytic ESCRT-0 complex, *in vivo*, through the genetic ablation of its two main components Hrs and Stam, at different steps of the *Drosophila* development. We show that the loss of Hrs and Stam produce similar phenotypes in all tissues examined, indicating that these proteins mainly function as a single complex throughout development. Our results indicate that Stam and Hrs participate in the efficient activation of the FGFR pathway in larval tracheal cells. In contrast, the ESCRT-0 acts to attenuate the extent of EGFR activation during embryogenesis. However, we find that Stam and Hrs are also required for the expression of EGFR targets in the adult wing. These data therefore support the view that the ESCRT-0 complex mediates directly or indirectly both positive and negative regulation of RTK signaling, depending on developmental contexts.

### *stam* and *hrs* are positive regulators of FGFR signaling

Tracheal cell migration in the air sac primordium (ASP) is controlled by FGFR signaling [29], [33]. Indeed, tracheal cells are not able to populate the distal tip of the ASP when FGF signal reception or transmission is defective (in *btl* mutant cells, for instance) or when cells experience too much FGF signaling [33]. We show here that *stam* and *hrs* are required for tracheal cells to efficiently colonize the tip of the ASP. Consistent with a role of Hrs and Stam in endosomal cargo sorting, we found that Btl/FGFR receptors accumulate in enlarged cytoplasmic vesicles in *stam* and *hrs* mutant cells, whereas they accumulate at the plasma membrane in wild type cells. Rab5-positive vesicles corresponding to early endosomes were also found to be enlarged in the absence of *hrs* and *stam*, suggesting that Btl/FGFR accumulates in altered early endosomes in mutant cells.

To test whether FGFR signaling is either enhanced or diminished in the absence of *stam*, we monitored the expression of a *pnt-lacZ reporter* gene and found that *stam* is required for its transcriptional activation, and hence most probably for high levels of FGFR signaling. These data show that the total level of FGF activity in tracheal cells is essential for efficient cell migration, which is in contrast with border cell migration, where localized signaling activity in cell is necessary for migration and not general level [22]. In order to further investigate the role of Hrs and the Hrs/Stam complex in FGFR signaling, we analyzed the effect of these mutations in the formation of projections by tracheal TCs, another cellular process which requires FGFR signaling. It has previously been shown that the number of branches formed by terminal cells increases with increasing FGFR signaling activity [39]. We found that *stam* and *hrs* are required in terminal cells to form branches efficiently. In addition, *hrs* and *stam* genetically interact with FGFR signaling during this process, to promote the formation of terminal cell projections. Therefore, both in TC and in ASP cells, our data demonstrate that *hrs* and *stam* are positive regulators of FGFR signaling in the *Drosophila* larval tracheal system.

Interestingly, it has been previously reported that *awd/Nm23* (*abnormal wing disc*) and *shibire*/*dynamin* down-regulate FGF signaling during embryonic tracheal development through endocytosis of Btl/FGFR [43]. It is therefore possible that the ESCRT-0 complex might also act to attenuate FGFR signaling during tracheal development in embryos, albeit the early lethality of embryos lacking Stam or Hrs prevents this hypothesis to be tested experimentally. Alternatively, different machineries regulating endocytosis and eventual endosome sorting might display antagonistic activity toward FGF signaling. For example, work in mammalian cell culture has shown that Rab5, a key component of the early endocytic pathway, colocalizes with activated FGFRs and that the depletion of *rab5* decreases FGFR signaling [44]. Finally, it has been shown that two different FGF ligands, KGF (Keratinocyte Growth Factor) and FGF10, can target the FGFR to the degradation pathway or the recycling pathway, respectively [45]. These results show that early endocytosis is critical for fine tuning of FGFR signaling activity; depending on the context, different endocytic mechanisms might differentially regulate FGFR signaling.

### Dual function of *stam* and *hrs* in EGFR signaling

The role of the ESCRT-0 complex in regulating the degradation of EGFR receptors has been well studied in cultured cells [10], [46]. Functional assays using RNA interference have shown that Hrs and Stam trigger the sorting of ubiquitinated receptors and downregulate EGFR signaling activity [10]. During *Drosophila* embryogenesis, *hrs* was also well established as a negative regulator of signaling RTK pathways such as EGFR and Torso [17]. Our recent isolation of the first mutations of the *stam* gene in flies [23] allowed us to show, here, that *stam* attenuates the EGFR activity during gastrulation and formation of tracheal placodes, suggesting that Stam and Hrs likely function within a common ESCRT-0 complex during embryogenesis.

Having shown that EGFR signaling is strongly upregulated in the absence of *hrs* and *stam* in the embryo, we tested whether EGFR signaling was also over-activated in the pupal wing upon reduction of *stam* and *hrs* activity. Assuming that *argos* expression in veins represents a faithfull readout of EGFR signaling, we observed instead a loss of EGFR activity in the absence of *hrs* and *stam* at the pupal stage. Supporting our conclusions, it was recently reported that *hrs* is also required for EGFR signaling at earlier stages, during the patterning of larval discs [20]. Therefore, these data provide independent evidence that, contrary to what was observed during embryogenesis, *hrs* and *stam* are necessary for high level EGFR signaling during wing development, from larval to pupal stages.

### Concluding remarks

We show that *stam* and *hrs* modulate RTK signalling activities either positively or negatively according to the tissue and the developmental stage. *hrs* and *stam* are essential for efficient activation of the FGFR signalling in the tracheal system, *e.g.*, they may promote recycling of the FGFR/Btl receptor to the plasma membrane. In addition, *hrs* and *stam* contribute to either enhance or attenuate EGFR signalling activity, depending on the tissue context. Similar to what has been proposed for FGFR signalling, positive versus negative modulation of EGFR activity may depend on the ligand which activates the receptors. Whereas Spitz is the only known EGFR ligand acting during early embryogenesis, Keren could be an additional ligand with Spitz to activate EGFR during vein differentiation [47], [48]. Another possible explanation for the tissue-specific antagonistic activities of the ESCRT-0 complex concerns the phosphorylation state of its main components. Depending of the amino acid residues which are phosphorylated in the Hrs and Stam proteins, their influence on the modulation of signalling activity vary [49]. Modulation of the phosphorylation code of Stam and Hrs between tissues may turn those molecules into pathway-specific activators or repressors.

Our compared analysis of the individual roles of Hrs and Stam *in vivo* strongly support that these two proteins act together to modulate RTK signalling, *i.e.*, within the ESCRT-0 complex. Regarding endosome maturation in Garland cells, our analyses show, however, that *hrs* and *stam* mutant cells do not display identical phenotype in this process; endosomes being much larger in the absence of *stam* than in the absence of *hrs* (or in the absence of both *stam* and *hrs*). Assuming that available mutations for both Stam and Hrs correspond to null alleles, which is fully compatible with their molecular nature, it remains possible that these different roles on endosome maturation may be linked to distinct protein partners of Stam and Hrs. The availability of *stam* loss of function mutations and rescuing transgenes, provide novel tools that will allow further dissection of the Stam protein function *in vivo*, and ultimately help in gaining further insights into the role of endocytosis during development.
